# Prostaglandin E2 receptors in asthma and in chronic rhinosinusitis/nasal polyps with and without aspirin hypersensitivity

**DOI:** 10.1186/s12931-014-0100-7

**Published:** 2014-08-26

**Authors:** Liliana Machado-Carvalho, Jordi Roca-Ferrer, César Picado

**Affiliations:** Immunoal · lèrgia Respiratòria Clínica i Experimental, CELLEX, Institut d’Investigacions Biomèdiques August Pi i Sunyer (IDIBAPS), Casanova 143, 08036 Barcelona, Spain; Servei de Pneumologia i Al · lèrgia Respiratòria, Hospital Clínic, Universitat de Barcelona, Barcelona, Spain; Centro de Investigaciones Biomédicas en Red de Enfermedades Respiratorias (CIBERES), Instituto de Salud Carlos III, Madrid, Spain

**Keywords:** Aspirin exacerbated respiratory disease, Asthma, Chronic rhinosinusitis, Nasal polyps, Prostaglandin E_2_, Prostaglandin E_2_ receptors

## Abstract

Chronic rhinosinusitis with nasal polyps (CRSwNP) and asthma frequently coexist and are always present in patients with aspirin exacerbated respiratory disease (AERD). Although the pathogenic mechanisms of this condition are still unknown, AERD may be due, at least in part, to an imbalance in eicosanoid metabolism (increased production of cysteinyl leukotrienes (CysLTs) and reduced biosynthesis of prostaglandin (PG) E_2_), possibly increasing and perpetuating the process of inflammation. PGE_2_ results from the metabolism of arachidonic acid (AA) by cyclooxygenase (COX) enzymes, and seems to play a central role in homeostasis maintenance and inflammatory response modulation in airways. Therefore, the abnormal regulation of PGE_2_ could contribute to the exacerbated processes observed in AERD. PGE_2_ exerts its actions through four G-protein-coupled receptors designated E-prostanoid (EP) receptors EP1, EP2, EP3, and EP4. Altered PGE_2_ production as well as differential EP receptor expression has been reported in both upper and lower airways of patients with AERD. Since the heterogeneity of these receptors is the key for the multiple biological effects of PGE_2_ this review focuses on the studies available to elucidate the importance of these receptors in inflammatory airway diseases.

**Electronic supplementary material:**

The online version of this article (doi:10.1186/s12931-014-0100-7) contains supplementary material, which is available to authorized users.

## Introduction

The purpose of this review is to offer a global overview of the significant literature available about prostaglandin (PG) E_2_ receptors in asthma, chronic rhinosinusitis (CRS) and nasal polyposis, with and without aspirin hypersensitivity.

### Asthma

Pathophysiologically, asthma is a multifactorial and complex chronic inflammatory disorder of the lung and is characterized by epithelial disruption, airway smooth muscle hypertrophy and hyperplasia, increased mucus secretion, basement thickening, increased cytokine production and chronic infiltration of inflammatory cells [1,2]. Depending on the severity of the disorder, it manifests clinically with repeated, variable, and episodic attacks of cough, wheezing and breathlessness [3,4]. The most effective drugs used in asthma control are inhaled corticosteroids. Although recommended and clinically effective in most asthma patients, airway remodelling changes can be resistant to the conventional pharmacological approach [5]. Various factors can trigger and/or develop asthma attacks: allergens, exercise, cold exposure, chemical sensitizers, air pollutants, and respiratory viral infections [3]. Conventionally, classification into atopic and nonatopic asthma is based on the presence or absence of clinical symptoms precipitated by one or more allergens. The presence of allergen-specific antibodies can be identified by skin prick testing or by measuring the level of specific immunoglobulin (Ig) E in serum [6,7].

Airway inflammation in asthma is associated with a massive influx of inflammatory and immune cells within the airways, including eosinophils, T helper (Th) 2 lymphocytes, mast cells, neutrophils and macrophages. Local overproduction of Th2 cytokines such as interleukin (IL)-4, IL-5, IL-9 and IL-13 by Th2 cells plays an important role in its pathophysiology [6]. IL-4 promotes Th2 cell differentiation, induces IgE production and increases IgE receptors; IL-5 is responsible for promoting eosinophil development, differentiation, recruitment, activation and survival; finally, IL-13 mediates allergen-induced airway hyperresponsiveness [8].

### Chronic rhinosinusitis and nasal polyposis

According to the *European Position Paper on Rhinosinusitis* (EPOS) [9] rhinosinusitis is defined as an inflammatory process of the nose and the paranasal sinuses characterized by two or more symptoms: nasal blockage/obstruction/congestion or nasal discharge, as well as facial pain/pressure or reduction/loss of smell [9]. This disorder may be classified into two forms, according to the duration of the symptoms, as acute or chronic [9–11]. In fact, the chronic form that persists beyond 12 weeks without complete resolution is associated with a lower quality of life and constitutes one of the most common health care problems [12]. CRS is subdivided itself into CRS with or without nasal polyps (NPs). Chronic rhinosinusitis with nasal polyps (CRSwNP) is a clinical phenotype found in up to 4% of the population [13]. The condition consists of loose connective tissue, oedema, inflammatory cells, and some glands and capillaries leading to nasal obstruction, secretion, loss of smell and headache [14]. Although the eosinophils are the most common cells in NPs, other cell types are also present, such as neutrophils, mast cells, plasma cells, lymphocytes, monocytes and fibroblasts [15,16].

### Aspirin exacerbated respiratory disease

Aspirin exacerbated respiratory disease (AERD) is a clinical syndrome characterized by hypersensitivity to aspirin and other non-steroidal anti-inflammatory drugs (NSAIDs), bronchial asthma and CRS with recurrent NPs [17,18]. AERD affects 10-20% of the asthmatic patient population and 8-26% of those diagnosed with CRSwNP [19]. The ingestion of aspirin or other NSAIDs in these patients provokes bronchoconstriction and exacerbates bronchospasms with attacks of asthma and rhinitis [20]. Effectively the characteristic symptoms of this disorder include moderate to severe asthma, massive eosinophilic infiltration and high prevalence of CRS associated with nasal polyposis [21].

The pathogenic mechanism underlying this disorder is believed to involve, at least in part, alterations in the eicosanoid metabolism and altered eicosanoid receptor expression [22–24]. However, and despite all the efforts, the pathogenicity of AERD is still not fully understood.

### Arachidonic acid metabolism

Arachidonic acid (AA) is a 20-carbon polyunsaturated fatty acid and is the main precursor of eicosanoids, mediating important functions in homeostasis, inflammation and immunoregulation [25]. Under normal conditions AA is not freely available and its concentration within the cell is very low. The availability of free AA is essential for the biosynthesis of eicosanoids. Upon activation of the cell and by the action of various phospholipases (preferentially cytosolic phospholipase A_2_) AA is released from the membrane phospholipids [26]. Therefore, once released, AA is rapidly metabolized through three enzymatic pathways namely cyclooxygenase (COX), lipoxygenase (LO), and cytochrome oxidases (hydrolase, epoxygenase) and one non-enzymatic pathway [27].

### Lipoxygenase pathway

**Figure 1:**
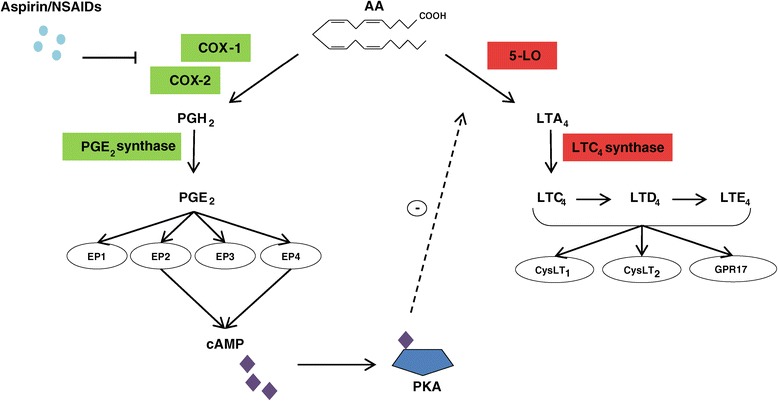
**Cyclooxygenase and 5-lipoxygenase pathways with special reference to aspirin exacerbated respiratory disease.** Simplified pictogram of biosynthetic pathways of prostaglandin (PG) E_2_ and cysteinyl leukotrienes (CysLTs). Arachidonic acid (AA) is metabolized by the cyclooxygenase (COX) or 5-lipoxygenase (5-LO) pathways. COX enzymes generate PGH_2_ which through PGE_2_ synthase is converted into PGE_2_. PGE_2_ couples to four subtypes of G-protein-coupled membrane receptors denominated E-prostanoid (EP) receptors. The activation of EP2 and EP4 receptors generates cyclic adenosine monophosphate (cAMP). This mediator negatively regulates the 5-LO pathway by activating protein kinase A (PKA). AA is also metabolized by the 5-LO pathway to generate leukotriene (LT) A_4_. LTA_4_ is subsequently metabolized by LTC_4_ synthase, which conjugates reduced glutathione into LTC_4_. LTC_4_ is metabolized into LTD_4_, which is then metabolized into LTE_4_. LTC_4_, LTD_4_ and LTE_4_ are designated as CysLTs. CysLTs bind to CysLT_1_, CysLT_2_ and GPR17 receptors. In aspirin exacerbated respiratory disease (AERD) the inhibition of COX pathway accounts for a reduced production of cAMP since the production of PGE_2_ is diminished. This is a simplified pictogram where some pathways and compounds generated are not mentioned.

AA, which is esterified on plasma membrane phospholipids, is released and converted into leukotriene (LT) A_4_ through 5-LO activity (Figure 1). LTA_4_ is subsequently converted by LTA_4_ hydrolase into LTB_4_ and by LTC_4_ synthase, which conjugates reduced glutathione into LTC_4_. This is metabolized into LTD_4_, which is then metabolized into LTE_4_. LTC_4_, LTD_4_ and LTE_4_ are designated as cysteinyl leukotrienes (CysLTs). LTs are synthesized upon cellular activation and the intracellular expression and distribution of 5-LO varies considering the cell type. In the airways, 5-LO is present in several types of leukocytes and becomes activated during allergic inflammation [8]. CysLTs activate three receptors with differential selectivity (CysLT_1_, CysLT_2_ and GPR17) and the stimulation of these receptors, principally CysLT_1_, account for most of its effects [21]. This receptor is expressed in a large variety of cells which include monocytes, macrophages, eosinophils, basophils, mast cells, neutrophils, T cells, B lymphocytes, pluripotent hematopoietic cells, interstitial cells of the nasal mucosa (NM), airway smooth muscle cells, bronchial fibroblasts and vascular endothelial cells [28] and its activation contributes to most of the effects of CysLTs that are relevant to the pathophysiological changes observed in patients with asthma [8].

### Cyclooxygenase pathway

AA can be metabolized through the COX pathway by the action of the COX enzymes (Figure 1). COX is a bifunctional enzyme with two active sites, one with COX activity that catalyzes the reduction of AA to form PGG_2_ and the other with peroxidase activity involved in the reduction of peroxidase group in PGG_2_ to hydroxyl group forming PGH_2_. These enzymes catalyze the reactions responsible for the production of several bioactive prostanoids, such as PGs, prostacyclins and thromboxane [29]. Studies show the presence of two isoforms of COX enzymes, namely COX-1 and COX-2. COX-1, the dominant source of prostanoids that serves a number of physiologic “housekeeping” functions, presents a uniform expression in almost all tissue and is generally considered constitutive [30]. COX-2 is described, in diverse studies, as highly induced and only expressed in response to certain inflammatory stimuli [31–35]. Furthermore, COX-2 is the more important source of prostanoid formation in inflammatory processes and proliferative diseases [36]. Both isoforms are located in the endoplasmatic reticulum and nuclear envelope, COX-2 is more highly concentrated in the nuclear envelope [37]. PGE_2_ is one of the most abundant prostanoids produced in the body [36]. Montuschi and co-workers [38] evaluated the effects of COX inhibition on exhaled eicosanoids in patients with chronic obstructive pulmonary disease. The authors found that in exhaled breath condensate, PGE_2_ is primarily derived from COX-1 activity. PGE_2_ exhibits pleiotropic and contrasting effects in different cell types and organs. According to Vancheri *et al*. [39] the lung is considered a privileged site for the action of PGE_2_. Although in this organ PGE_2_ can exert anti-inflammatory, anti-fibrotic and immune restrictive actions, it can also mediate pro-inflammatory responses [39,40].

### Prostaglandin E_2_ receptors

The activity of PGE_2_ is mediated by a group of rhodopsin-type G-protein-coupled membrane receptors (GPCRs) denominated E-prostanoid (EP) receptors [41]. There are four GPCR subtypes: EP1, EP2, EP3 and EP4. The physiological and contrasting effects of PGE_2_ depend on the expression or the co-expression of more than one receptor or isoform [42]. Additionally, each receptor may be differentially expressed in tissues. EP receptors differ in terms of intracellular signalling [43,44]. These receptors could be classified according to their intracellular signalling and second messenger [45]. The EP1 receptor signals via Ca^2+^ mobilization with slight phosphatidylinositol activity [46,47]. Distribution of this receptor in human tissues and cells is restricted and has been demonstrated in the myometrium, pulmonary veins, mast cells, colonic longitudinal muscle and keratinocytes. EP1 exerts mostly constrictive functions, however and compared with other prostanoid receptors, it seems to be less studied [47]. EP2 and EP4 receptors increase intracellular cyclic adenosine monophosphate (cAMP) through activation of adenyl cyclase [39,48]. Functional studies have demonstrated that the EP2 receptor is widely distributed [49] and it seems to be involved in processes of relaxation such as bronchodilation [50] and anti-inflammation [51]. On the other hand, EP4 is also widely distributed [44]. In a direct comparison with EP2 receptor subtype signalling, the EP4 receptor demonstrated a less efficient functional coupling to cAMP [52,53]. Effectively, studies have reported several cAMP-independent signalling pathways for EP4 receptor activation [54–59]. EP4 mediates vasorelaxation of pulmonary arterial veins and also promotes anti-inflammatory effects [60]. Consistent with its bronchoprotective action, PGE_2_ inhibited proliferation and migration of bronchial smooth muscle cells through the action of the EP4 receptor [61]. Studies have revealed that the EP3 receptor shows a wide distribution in almost all tissues and consists of multiple isoforms generated by numerous alternative splicing of the C-terminal [62–64]. Signalling through this receptor reduces the levels of cAMP and increases intracellular Ca^2+^ [44]. EP3 exerts mainly contractile functions such as the constriction of human pulmonary artery in the lung [65]. Moreover, this receptor has been implicated in inflammation, pain and cough [47,66].

In summary, EP2 and EP4 receptor signalling promotes the accumulation of cAMP which is normally related to inhibition of cell functions. On the other hand, EP1 and EP3 receptors that increase intracellular calcium could be associated with cellular activation.

### Arachidonic acid metabolism in aspirin exacerbated respiratory disease

The pathogenesis of AERD is not fully understood but several studies have reported that the pathogenic mechanisms of this condition may be due, at least in part, to marked imbalance in eicosanoid metabolism possibly increasing and perpetuating the process of inflammation [20,22,67]. Aspirin and other NSAIDs block the COX pathway by acetylation of the COX enzyme and consequently inhibit conversion of AA to PG. The dependency on COX products to modulate and maintain homeostasis over 5-LO activity is a unique feature of AERD [68]. Effectively in this disorder the inhibition of COX-1 results in the overproduction of CysLTs. CysLTs are potent bronchoconstrictors that contribute to the pathophysiological changes observed in patients with asthma. CysLTs, by increasing pulmonary microvascular permeability and mucus hypersecretion can contribute to bronchial obstruction in asthmatic patients [69–72]. Diamant *et al*. [73] reported that the inhalation of CysLTs increases eosinophilia in sputum of asthmatic patients and induces the recruitment of eosinophils into the airway mucosa. Indeed, CysLTs might participate in the process of airway remodeling, including eosinophilic airway inflammation, airway smooth muscle cell hyperplasia, mucus gland hyperplasia, mucus hypersecretion, and fibrous collagen depositions [69,70]. Several studies have described the biological effects and the contribution of these lipid mediators in AERD. After challenging AERD patients with oral, intravenous and intranasal aspirin treatments, the levels of CysLTs are significantly increased [74,75]. Moreover, patients with AERD excrete higher levels of LTE_4_ in their urine when compared with asthmatic patients without aspirin intolerance [76,77]. Pérez-Novo *et al*. [78] reported that the nasal tissue of patients with CRSwNP presents elevated levels of CysLTs when compared with NM from aspirin-tolerant (AT) asthmatic patients and this increased production is associated with the elevated expression of LTC_4_ synthase, the terminal enzyme in the production of CysLTs. In summary, the overproduction of CysLTs reported in AERD seems to play an important role in the pathogenesis of the disease, since the levels of this mediator are comparatively reduced in asthmatic patients without aspirin intolerance.

PGE_2_ formed from COX-dependent conversion of AA have demonstrated inhibitory effects on CysLTs production. PGE_2_ administration blocks bronchoconstriction and inhibits the increase in urinary LTE_4_ that occur with aspirin challenge in subjects with AERD [79,80]. Pharmacological studies suggest that most urinary PGE_2_ metabolites in AT asthmatic patients and healthy subjects derive from COX-2 [81]. Several studies have demonstrated that NP tissue from subjects with or without aspirin sensitivity shows impaired expression of COX-2 [82,83] and hypermethylation of the PGE_2_ synthase *(PTGES)* gene in patients with AERD when compared with polyps from AT patients [84]. Moreover, experiments in *vitro* have shown a reduced production of PGE_2_ in epithelial cells from NPs [85], bronchial fibroblasts [86], and peripheral blood leukocytes from patients with AERD [87]. The combination of a reduced expression of COX-2 in inflammatory conditions in subjects with AERD with hypermethylation of the *PTGES* gene reported could be responsible for the low production of PGE_2_ observed in these subjects. Thereby, in patients with AERD, when COX-1 is inhibited by aspirin or other NSAIDs, the diminished availability of PGE_2_ will contribute to the exacerbations of the characteristic symptoms of this pathology.

### Prostaglandin E_2_ receptors in asthma and chronic rhinosinusitis with nasal polyps

Few studies have been performed to elucidate the importance of this receptor family in upper and lower respiratory airway diseases. The literature mainly describes the expression and cellular distribution of these receptors at different levels of the airways, using for that purpose diverse type of samples, such as biopsies, cultured cells or peripheral blood and different techniques.

#### Asthma

Additional file 1: Table S1 shows the main publications on EP receptors in asthma [see Additional file 1: Table S1]. Ying and co-workers [88] used immunocytochemistry to compare the expression and cellular distribution of the EP receptors in induced sputum cells from asthmatic patients and control subjects. They reported that sputum cells showed immunoreactivity for all receptors in both patients with asthma and control subjects. However, in patients with asthma, they found a high immunoreactivity for EP2 and EP4, but not EP1 and EP3 receptors on macrophages when compared with control subjects. The investigators concluded that the pattern of EP receptor expression is particularly increased in airway macrophages of patients with asthma.

In a study performed with bronchial biopsies [89] from both AERD and AT asthmatic patients and control subjects, the authors reported that, compared with AT, patients with AERD have increased bronchial mucosal neutrophil and eosinophil numbers but reduced percentages of T cells, macrophages, mast cells and neutrophils expressing EP2. In contrast, quantitative analysis of EP receptor mRNA expression in peripheral blood mononuclear cells isolated from these patients showed no significant differences between the two groups.

#### Chronic rhinosinusitis and nasal polyps

In Additional file 2: Table S2 we highlight the studies that have been performed to elucidate the importance of the EP receptors on the upper airways [see Additional file 2: Table S2]. Pérez-Novo *et al*. [90] studied the possible link between the expression of prostanoid receptors and the eosinophilic inflammation characteristic of paranasal sinus diseases by means of real-time PCR. The results of this study showed a high mRNA expression of EP2 and EP4 receptors in nasal tissue from both CRS without NP (CRSsNP) and CRSwNP patients when compared with control subjects. On the other hand, EP1 and EP3 receptors seem to be downregulated in nasal tissue from CRSwNP when compared with CRSsNP patients and control subjects.

Ying and co-workers [91], in a study based on immunohistochemistry, analysed the expression pattern of EP receptors in nasal biopsies from CRSwNP patients with AERD and AT and control subjects. The extensive analysis showed that, globally, mucosal expression of EP1 and EP2, but not EP3 and EP4 was significantly elevated in nasal biopsies from both patients with AERD and AT patients when compared with nasal biopsies extracted from control subjects. The researchers attribute the results principally to the high percentage of epithelial cells and goblet cells expressing these receptors. In inflammatory cells, the findings reported were different. They showed that the percentages of neutrophils, mast cells, eosinophils and T cells expressing EP2, but not EP1, EP3, or EP4, were significantly reduced in AERD patients when compared with AT patients.

Effectively, EP2 receptor downregulation seems to be common in both upper and lower airways of patients with AERD. Adamusiak and collaborators [92] described that, in NPs from AERD patients, the density of cells expressing the EP2 receptor was significantly lower when compared with NP from AT patients.

Using the Western blot technique, Roca-Ferrer *et al*. [83], using fibroblast cell cultures isolated from NP of both AERD and AT patients, described that there were low levels of EP2 protein receptor expression under inflammatory conditions when compared with fibroblasts isolated from NM of control subjects.

Apart from these expression studies, various polymorphisms in EP2 gene (*PTGER2*) were described [93,94] and they could possibly be related to AERD. In an extensive candidate gene analysis study to identify susceptibilities to AERD in the Japanese population, Jinnai and co-workers [93] showed the association of AERD with a functional single-nucleotide polymorphism in *PTGER2* that decreases the transcription level of the receptor *in vitro*.

As previously described, the ability of PGE_2_ to induce or suppress various mechanisms involved in inflammatory processes indicates the complex activities of its receptor. The activation of EP2 and EP4 (Figure 1) initiates the production of cAMP, a secondary messenger that acts by activating protein kinase A (PKA). Once activated, PKA has the capacity to regulate 5-LO [95,96] by phosphorylation of serine-523 in 5-LO, suppressing its function. Effectively, Luo and co-workers [96] described that the mutation of serine-523 on human 5-LO prevents phosphorylation by PKA and promotes the abnormal synthesis of LTs. The dysfunctional signalling through cAMP and PKA contributes to a variety of diseases, including those characterized by chronic inflammation. In 1983, Ham and colleagues [97] showed for the first time that PGE_2_ inhibited LT biosynthesis in activated neutrophils, and the inhibition was mediated by an increment of cAMP levels. Indeed, the mechanisms by which the enhancement of cAMP (by PGE_2_ or other cAMP-elevating agents) are able to down-regulate LT biosynthesis involve the inhibition of the translocation of 5-LO to the nuclear envelope in human polymorphonuclear leukocytes [80]. The mechanisms and receptors by which PGE_2_ modulates the activation of human mast cells have also been assessed [95]. The investigators described that PGE_2_ can attenuate through EP2 receptors the generation of CysLTs in activated mast cells. The effect of low levels of EP2 on the downstream signalling pathway as well as the polymorphic variants of its gene are still unclear and further studies are needed to determine the functional repercussion of these alterations. Nevertheless, considering the regulatory effect of PGE_2_ on 5-LO through EP2 receptors these alterations could contribute, at least in part, to the exacerbation of the inflammatory processes demonstrated in patients with AERD.

### Targeting prostaglandin E_2_ receptors

All the available information about the role of EP receptor subtypes in inflammatory airway diseases or in a totally different disorder comes partly from genetic ablation of prostanoid receptors or from studies performed with selective EP receptor agonists or antagonists. The latter strategy includes the development of small-molecule ligands that target a specific EP receptor and whose purpose is receptor inhibition or activation. All EP receptors are activated by their natural agonist PGE_2_ or by a number of PGE_2_ analogues named agonists and inhibited by antagonists. Over the years several studies have been performed to develop these compounds, which are used to find a possible therapeutic approach to treat numerous diseases [47].

As described previously, the EP2 receptor exerts many inhibitory functions. PGE_2_ has been considered to be a bronchodilator and anti-inflammatory natural substance with potential for treating asthma and other respiratory diseases. The use of developed selective agonists improved this viewpoint. Effectively, the bronchodilator effect of PGE_2_ is mediated by the EP2 receptor, which promotes airway relaxation and inhibits IgE-dependent mast cell activation [98]. Recent studies have also shown that the EP4 receptor mediates bronchodilation supporting the idea that targeting this receptor may be a novel therapeutic approach for obstructive airway diseases [99]. Patients with AERD characteristically showed a critical deficiency in PGE_2_/EP2 signalling. Considering the protective and beneficial effects of the PGE_2_/EP2 axis in airways, the use of specific commercially developed agonists could correct this deficit and ameliorate the inflammation scenario in these patients. The potential use of inhibitors of the EP3 receptor in the treatment of chronic cough has also been recently proposed [66].

## Conclusions

Differential regulation and expression patterns of PGE_2_ receptors were observed in each of the chronic inflammatory airway diseases presented in this review. Although these alterations may worsen the already diminished levels of PGE_2_, additional studies are necessary to reveal further information about the role of these receptors in asthma and CRS with or without NPs or aspirin hypersensitivity. Moreover, EP receptors represent potential targets for therapeutic approaches. The use of PGE_2_ analogues and synthetic drugs, which can selectively and specifically agonize or antagonize signalling from EP receptor subtypes, has proved very useful for a deeper understanding of the pathologic mechanisms where PGE_2_ and its receptors are involved.

## Additional files

Additional file 1: Table S1. Prostaglandin E_2_ receptor expression in lower airways. Additional file 2: Table S2. Prostaglandin E_2_ receptor expression in upper airways.

## Abbreviations

AA: Arachidonic acid
AERD: Aspirin exacerbated respiratory disease
AT: Aspirin-tolerant
cAMP: Cyclic adenosine monophosphate
COX: Cyclooxygenase
CRS: Chronic rhinosinusitis
CRSwNP: Chronic rhinosinusitis with nasal polyp
CRSsNP: Chronic rhinosinusitis without nasal polyp
CysLTs: Cysteinyl leukotrienes
EP: E-prostanoid
GPCR: G-protein-coupled membrane receptor
Ig: Immunoglobulin
IL: Interleukin
LO: Lipoxygenase
LT: Leukotriene
NM: Nasal mucosa
NP: Nasal polyp
NSAID: Non-steroidal anti-inflammatory drug
PG: Prostaglandin
PKA: Protein kinase A
Th: T helper
