# Supplementary material for: Prostaglandin E2 receptors in asthma and in chronic rhinosinusitis/nasal polyps with and without aspirin hypersensitivity
Source: Respir Res. 2014 Aug 26;15(1):100. doi: 10.1186/s12931-014-0100-7 (PMC4243732; doi:10.1186/s12931-014-0100-7)
Supplement: Additional file 1: Table S1. — Prostaglandin E2 receptor expression in lower airways. [file 12931_2014_100_MOESM1_ESM.doc]

**Additional file 1: Table S1. Prostaglandin E_2_ receptor expression in lower airways**

| **Study reference** | **Sample** | **Measurement** | **Technique** | **EP1 expression** | **EP2 expression** | **EP3 expression** | **EP4 expression** |
| --- | --- | --- | --- | --- | --- | --- | --- |
| [89] | Bronchial biopsies from control subjects;  Bronchial biopsies from AERD patients;  Bronchial biopsies from AT asthmatic patients | EP receptor protein expression in whole tissue | IHC | **Global expression:** high in bronchial biopsies from both AERD and AT asthmatic patients compared with bronchial biopsies from control subjects  **Substructural expression:** high in the bronchial epithelium of both AERD and AT asthmatic patients compared with control subjects  **Submucosa expression:** high in both AERD and AT asthmatic patients compared with control subjects  **Expression on inflammatory cells:** high on T-cells, macrophages, eosinophils, and neutrophils in bronchial biopsies from both AERD and AT asthmatic patients compared with control subjects | **Global expression:** high in bronchial biopsies from both AERD and AT asthmatic patients compared with bronchial biopsies from control subjects  **Substructural expression:** high in the bronchial epithelium of both AERD and AT asthmatic patients compared with control subjects  **Submucosa expression:** high in both AERD and AT asthmatic patients compared with control subjects  **Expression on inflammatory cells:** low on T-cells, macrophages, mast cells, and neutrophils in bronchial biopsies from AERD patients compared with AT asthmatic patients | **Global expression:** No differences  **Substructural expression:** No differences  **Submucosa expression:** high in AERD patients compared with control subjects  **Expression on inflammatory cells:** No differences | **Global expression:** No differences  **Substructural expression:** No differences  **Submucosa expression:** No differences  **Expression on inflammatory cells:** high on eosinophils in bronchial biopsies from both AERD and AT asthmatic patients when compared with control subjects |
| [88] | Sputum from control subjects;  sputum from asthmatic patients | EP receptor protein expression on sputum cells | ICC | No differences | High on macrophages in patients with asthma compared with control subjects | No differences | High on macrophages in patients with asthma compared with control subjects |

**Abbreviations:**

AERD: aspirin exacerbated respiratory disease

AT: aspirin- tolerant

EP: E-prostanoid

ICC: immunocytochemistry

IHC: immunohistochemistry
