# Supplementary material for: Prostaglandin E2 receptors in asthma and in chronic rhinosinusitis/nasal polyps with and without aspirin hypersensitivity
Source: Respir Res. 2014 Aug 26;15(1):100. doi: 10.1186/s12931-014-0100-7 (PMC4243732; doi:10.1186/s12931-014-0100-7)
Supplement: Additional file 2: Table S2. — Prostaglandin E2 receptor expression in upper airways. [file 12931_2014_100_MOESM2_ESM.doc]

**Additional file 2: Table S2. Prostaglandin E_2_ receptor expression in upper airways**

| **Study reference** | **Sample** | **Measurement** | **Technique** | **EP1 expression** | **EP2 expression** | **EP3 expression** | **EP4 expression** |
| --- | --- | --- | --- | --- | --- | --- | --- |
| [92] | NP from AT patients with CRSwNP;  NP from AERD patients with CRSwNP | EP receptor protein expression in whole tissue | IHC | Not performed | Low immunoreactivity in NP from AERD CRSwNP compared with NP from AT CRSwNP | Not performed | No differences |
| [90] | NM from control subjects  NM from patients with CRSsNP;  NM from patients with CRSwNP | EP receptor mRNA expression in whole tissue;  EP receptor protein expression in whole tissue | Real-time PCR;  IHC | Low mRNA expression in CRSwNP compared with both control subjects and CRSsNP | High mRNA expression in both CRSwNP and CRSsNP compared with NM from control subjects  High immunoreactivity in inflammatory cells compared with epithelium in CRSwNP | Low mRNA expression in CRSwNP compared with both NM from control subjects and CRSsNP | High mRNA expression in both CRSwNP and CRSsNP compared with NM from control subjects  High immunoreactivity in inflammatory cells compared with epithelium in CRSwNP |
| [83] | NM from control subjects  NP from AT patients with CRSwNP;  NP from AERD patients with CRSwNP | EP receptor protein expression in primary fibroblast culture | WB | No differences | Low stimulation under inflammatory conditions in both NP from AERD and AT CRSwNP compared with NM from control subjects | No differences | No differences |
| [91] | NM from control subjects;  NM from AT patients with CRSwNP;  NM from AERD patients with CRSwNP | EP receptor protein expression in whole tissue | IHC | **Global expression:** High in both NM from AERD and AT CRSwNP patients compared with NM from control subjects  **Expression on structural cells:** high on tubulin-β+ ciliated columnar epithelial cells in NM from both AERD and AT CRSwNP patients compared with NM from control subjects  **Expression on inflammatory cells:** no differences | **Global expression:** High in both AERD and AT CRSwNP compared with NM from control subjects  **Expression on structural cells:** high on tubulin-β+ ciliated columnar epithelial cells in NM from both AERD and AT CRSwNP patients compared with NM from control subjects  **Expression on inflammatory cells:** low on neutrophils, mast cells, eosinophils, and T-cells of NM from AERD CRSwNP patients compared with NM from AT CRSwNP patients | **Global expression:** No differences  **Expression on structural cells:** high on tubulin-β+ ciliated columnar epithelial cells in NM from both AERD and AT CRSwNP patients compared with NM from control subjects  **Expression on inflammatory cells:** no differences | **Global expression:** No differences  **Expression on structural cells:** no differences  **Expression on inflammatory cells:** no differences |

**Abbreviations:**

AERD: aspirin exacerbated respiratory disease

AT: aspirin-tolerant

CRSwNP: chronic rhinosinusitis with nasal polyp

CRSsNP: chronic rhinosinusitis without nasal polyp

EP: E-prostanoid

IHC: immunohistochemistry

NM: nasal mucosa

NP: nasal polyp

PCR: polymerase chain reaction

WB: western blot
